# Magnetic Resonance Imaging Evaluation of Hemangioma Resection for Encephalofacial Angiomatosis (Sturge–Weber Syndrome) in Children under Intelligent Algorithm

**DOI:** 10.1155/2022/7399255

**Published:** 2022-04-08

**Authors:** Yini Lv, Guoan Liang, Hailing Fan, Jun Cheng, Panwei Xing, Lili Zhu

**Affiliations:** ^1^Department of Pediatrics, Taizhou Hospital of Zhejiang Province Affiliated to Wenzhou Medical University, Taizhou 317000, Zhejiang, China; ^2^Department of Pathology, Taizhou Hospital of Zhejiang Province Affiliated to Wenzhou Medical University, Taizhou 317000, Zhejiang, China; ^3^Department of Radiology, Taizhou Hospital of Zhejiang Province Affiliated to Wenzhou Medical University, Taizhou 317000, Zhejiang, China; ^4^Department of Plastic Surgery, Taizhou Hospital of Zhejiang Province Affiliated to Wenzhou Medical University, Taizhou 317000, Zhejiang, China

## Abstract

This study was aimed to evaluate the clinical efficacy of hemangioma resection in the treatment of infantile encephalofacial angiomatosis (Sturge–Weber syndrome, SWS) through magnetic resonance imaging (MRI) images, and intelligent algorithms were employed to process MRI images. A retrospective study of 45 children diagnosed with facial hemangioma admitted to hospital was conducted. Then, MRS images were acquired, and a mathematical model for MRI image denoising and reconstruction was constructed based on nonlocal similar block low-rank prior algorithms. The processing effect was assessed regarding the peak signal-to-noise ratio (PSNR) and structural similarity (SSIM). Finally, MRI images were collected to analyze the difference between the metabolites of N-acetylaspartic acid (NAA), creatine (Cr), choline (Cho), and their ratios in the lesions of the children before and after treatment. The improvement rate was analyzed through a twelve-month follow-up. The algorithm test results showed that compared with the classic K-singular value decomposition (K-SVD) denoising algorithm and the Sparse MRI reconstruction algorithm, the proposed algorithm processed MRI images more clearly and had more detailed information. The quantitative results showed that the PSNR and SSIM in the image processed by the algorithm proposed were remarkably large. The clinical treatment results showed that compared with those before treatment, the nCho level after treatment, the ratio of Cho/Cr and Cho/NAA were remarkably reduced, and the difference was remarkable (*P* < 0.05). The follow-up results showed that the considerable improvement rate was 88.89%, the postoperative organ remodeling rate was 17.78%, and the probability of reoperation was only 6.67%. In summary, the introduction of intelligent algorithms for denoising and reconstruction of MRI images can remarkably improve image quality and help doctors use image information to diagnose diseases and evaluate treatment effects. The hemangioma resection for the treatment of pediatric SWS had a high treatment improvement rate and was worthy of clinical adoption.

## 1. Introduction

Hemangioma is the most common benign soft tissue tumor in children and has the dual nature of tumor and malformation [1]. Hemangioma in children is essentially caused by vascular dysplasia during embryonic development. In addition, excessive/abnormal differentiation of blood vessels can cause vascular malformations, so it belongs to hamartoma [2]. Statistical data showed that the clinical prevalence rate of hemangioma has been as high as 3%–8%, and women are more than men, and they are mostly in the head and face, chest and back, and limbs [3]. Mixed hemangioma is a strong invasive hemangioma, mainly growing in the face and neck. Generally, it grows rapidly and spreads six months after birth, seriously damaging the normal tissues and making the eyelid, lip, and ear covered by hemangioma tissues [4, 5]. Hemangiomas are irregular in shape, prone to bleeding, necrosis, infection, and other problems, and can cause respiratory, eating, and hearing dysfunction, or even death or disability in serious cases [6].

At present, the clinical treatment methods for hemangioma mainly include surgical resection, injection of sclerotherapy, hormone injection, and laser therapy [7]. Imaging methods used for diagnosis and treatment evaluation of SWS include ultrasound, digital subtraction angiography (DSA), computed tomography (CT), and magnetic resonance imaging (MRI) [8]. DSA is considered as the gold standard for diagnosing vascular diseases of the head and neck. However, children need to receive a large amount of contrast agent injection during examination, which has the risk of causing adverse reactions and organ damage, so it is not recommended in clinical practice. Ultrasound cannot directly show the lesion range of hemangioma. MR image has the advantages of noninvasive, nonradiation, and high sensitivity, which is applied in the diagnosis and treatment of hemangioma [9]. However, the speed of MR is relatively slow in the imaging process, and slight movement will cause the generation of artifacts [10]. In the adoption of MR image, how to generate high spatial and temporal resolution MR image quickly has become an urgent problem to be solved. The denoising and reconstruction of MR images using intelligent algorithms can accurately recover the details of the image [11]. At present, intelligent algorithms have been widely applied in heart imaging, brain imaging, and other organ imaging, which have achieved excellent processing effects [12].

In this research, hemangioma resection was performed in children with SWS. MR images were collected before and after treatment, and MR images were denoised and reconstructed by intelligent algorithm. The therapeutic effect of the children after treatment was analyzed based on the processed MR images and related parameters. The results of this study were intended to provide reference for improving the therapeutic effect of children with SWS.

## 2. Materials and Methods

### 2.1. General Information

A retrospective study was conducted on 78 children diagnosed with SWSS admitted to hospital from January 2018 to March 2021, and 33 cases that did not meet the study conditions were excluded. A total of 45 patients were included in the study. There were 20 males and 25 females, ranging in age from 4 to 13 years, with an average age of 6.78 ± 2.67 years, and a medical history ranging from 1 month to 6 years, with an average history of 2.78 ± 1.69 years. The study had been approved by the ethics committee of hospital, and all the subjects included in the study had signed the informed consent.

Inclusion criteria: (i) those with complete medical records and image data; (ii) the facial area of children was larger and convex outward; (iii) palpated tumor volume diameter was 3–15 cm; (iv) the patient's skin was normal or dark red; (v) informed consent of patients and their families was acquired.

Exclusion criteria: (i) those who were complicated with major organ diseases such as heart, liver, and kidney; (ii) people with communication difficulties; (iii) those with hereditary or immune diseases; (iv) those who were complicated with other malignant tumors.

### 2.2. The Research Methods

**Figure 1:**
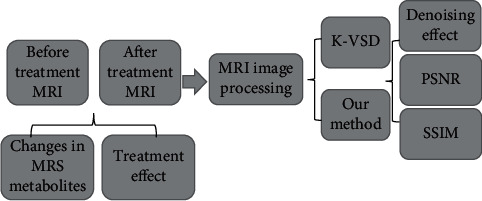
Flow chart of research method.

MRI scan was performed before and after treatment of cerebral hemangiomatosis in infants by hemangiectomy. The K-SVD algorithm and the algorithm proposed were used to denoise MRI images. The denoising effect was scored and compared with PSNR and SSIM of the two algorithms, and the therapeutic effect of hemangiectomy and changes of MRS metabolites were analyzed in Figure 1.

### 2.3. Magnetic Resonance Scanning

Conventional T1WI, T2WI, sagittal plane, and coronal plane T1WI sequences were scanned by 1.5 T MRI instrument and combined head and neck coil. MRS was scanned via cross-sectional 2D multivoxel MRS. The scan involved placing a saturation band at the cephalic end (the distal end of the blood flow) to block venous return signals to eliminate interference with venous flow. T1WI scan parameters were set as time of repetition of 450 ms and echo time of 9.5 ms. T2WI scan parameters were set as time of repetition of 3,500 ms and echo time of 90 ms. The scanning field was adjusted to fit the child's cranial size. All metal objects on the child were routinely removed, and oral chloral hydrate sedation was made according to the dosage of 50 mg/kg. Evaluation and analysis were conducted by two experienced radiologists.

### 2.4. Treatment Options

**Figure 2:**
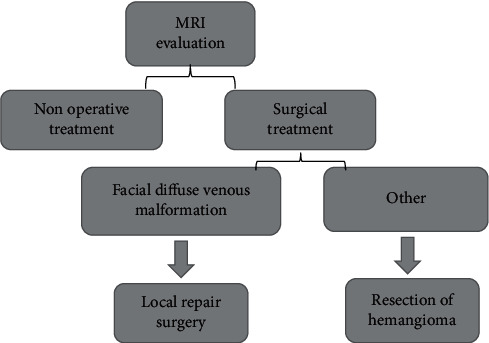
Flowchart of treatment plan.

Preoperative MRI was performed to evaluate the tumor area and adjacent relationship of surrounding tissues. According to the results of the evaluation, specific nonsurgical or surgical treatment was selected, and the corresponding surgical procedure was determined. For children with facial diffuse venous malformation, anhydrous alcohol and other measures were used to treat the tumor, and then local repair surgery was performed. Other children that can be treated with surgical resection were given hemangioma resection. A blood type test and blood preparation were required before surgery. For the tumor and its surrounding important tissues/organs that were relatively clear and the skin surface area of hemangioma that was relatively small, the skin was drawn and sutured after resection. After blunt dissection, the skin was completely resected. After tumor resection, local flap was used to cover the wound for repair (Figure 2).

### 2.5. MRI Image Processing Based on Intelligent Algorithm

#### 2.5.1. MRI Image Denoising

The nonlocal similar block low-rank prior algorithm is used to construct the denoising model of MRI images. The nonlocal mean algorithm mainly denoises the image through weighted average of pixels in similar image blocks [13]. It is assumed that the image with noise is *y* and the image domain is Ω, then any pixel in the image is *y*(*u*), *u* ∈ Ω, and the calculation equation for weighted average using nonlocal mean algorithm is the following equation.

(1) $$\begin{array}{c} NLM\left(y\left(u\right)\right)={\sum \omega \left(u,v\right)y\left(v\right)}^{},\;v\in \Omega^{\prime }. \end{array}$$

In the above equation, *u* is the pixel to be evaluated, Ω′ is the set of pixels similar to *u*, *ω* is the weight, and *ω* is determined by the similarity between the pixels.

The blocks centered on pixel *u* and pixel *v* are set as *T*_*u*_ and *T*_*v*_, respectively, and the similarity is calculated through the Euclidean distance.

(2) $$\begin{array}{c} D\left(u,v\right)={\left\|T_{u}-T_{v}\right\|}_{2,a}^{2}. \end{array}$$

In the above equation, *a* is the standard deviation of the Gaussian kernel function, and *a*>0.

The objective function based on the demand solution of the nonlocal low-rank denoising algorithm used in this study is the following equation.

(3) $$\begin{array}{c} \text{argmin rank}\left(Y_{u}\right),\\ \text{s.t. }{\left\|X-D\right\|}_{2}^{2}\leq \varepsilon ,\;\;u=1\cdots U. \end{array}$$

In the above equation, *D* is the image after denoising.

#### 2.5.2. MRI Image Reconstruction

A nonlocal similar block low-rank prior algorithm is used to construct a reconstruction model for MRI. The characteristics of the low-rank matrix are used to reconstruct the MRI images, and the following equation of the MRI reconstruction model is constructed in space *K*.

(4) $$\begin{array}{c} \text{arg }{\text{min}}_{x}\text{ rank}\left(B_{u}\right),\\ \text{s.t}.\;{\left\|A_{i}x-y\right\|}_{2}^{2}\leq \varepsilon ,\;\;u=1\cdots U. \end{array}$$

In the above equation, *B* is the similar block matrix of the reference image, *A*_*i*_*X* − *y*_2_^2^ is the fidelity term of the data, and *A* is the norm.

P2 algorithm is adopted to solve the above problem, and the corresponding objective function is the following equation.

(5) $$\begin{array}{c} x^{i+1}=\arg{\text{min}}_{x,B_{u}}{\left\|A_{i}x-y\right\|}_{2}^{2}+\varphi_{1}\sum_{u}{\left\|Q_{u}-P_{u}\right\|}_{A}^{2}. \end{array}$$

In the above equation, *φ* is a singular value.

The *A* value is written as the two-norm form of the vector, and the Fourier transform is performed to convert the image from the spatial domain to the Fourier domain. The equation is deformed, then the clustering of similar blocks in the MRI image is inversely processed, and the low-rank matrix is finally restored to the MRI image. The *A* value is written as the following equation.

(6) $$\begin{array}{c} Ax\left(u\right)=\left\{\begin{array}{cc} S\left(u\right) & \left(u\right)\notin \Omega \\ \frac{S\left(u\right)+vS_{0}\left(u\right)}{1+v} & \left(u\right)\in \Omega \end{array}.\right. \end{array}$$

In the above equation, *S*=*A*_*i*_^*u*+1^, *S*_0_=*vAA*_*j*_^*H*^*y*, and Ω is the down sampling area.

The acquired MRI images are analyzed to verify the performance of each model. The sampling rate of the reconstruction model is analyzed first. The calculation equation of the sampling rate (*R*) is the following equation.

(7) $$\begin{array}{c} R=\frac{\text{points obtained by sampling}}{\text{all data points}}. \end{array}$$

### 2.6. Image Quality Evaluation Indicators

- Evaluation of image denoising effect was that the evaluation process and criteria of images were determined by radiologists. Each image was professionally evaluated by three doctors in the radiology department with more than five years of clinical experience. The image denoising effect under different algorithms was scored, with a full score of 10. The higher the score, the better the denoising effect.
- Peak signal-to-noise ratio (PSNR) and structural similarity (SSIM) were used to evaluate image denoising and reconstruction performance. The higher the PSNR and SSIM, the more similar the image after denoising is to the original image, which indicates that the effect of maintaining the detailed information structure in the image is better, reflecting an excellent denoising effect of the algorithm. The calculation equations of PSNR and SSIM are as follows.

(8) $$\begin{array}{c} PSNR=10\log_{10}\frac{255^{2}}{1/mn\sum_{i=1}^{m}\sum_{j=1}^{n}{\left(x\left(i,j\right)-y\left(i,j\right)\right)}^{2}},\\ SSIM\left(x,y\right)={\left(\frac{2\mu_{x}\mu_{y}+C_{1}}{\mu_{x}^{2}+\mu_{y}^{2}+C_{1}}\right)}^{\alpha }{\left(\frac{2\sigma_{x}\sigma_{y}+C_{2}}{\sigma_{x}^{2}+\sigma_{y}^{2}+C_{2}}\right)}^{\beta }{\left(\frac{2\sigma_{x,y}+C_{3}}{\sigma_{x}^{2}\sigma_{y}^{2}+C_{3}}\right)}^{\gamma }. \end{array}$$

In the above equation, *x* and *y* are the amplitudes in different images, *μ* is the mean value of the image, *σ* is the standard deviation/covariance of the image, and *α*, *β*, and *γ* all equal to 1.

### 2.7. Efficacy Evaluation Indicators

MR scan was performed before and after treatment, and the MRS scan images were sent to the workstation. Functool II was employed for image processing, which automatically calculated metabolites N-acetylaspartic acid (NAA), creatine (Cr), choline (Cho), etc., and the standard nNAA, nCho, nCr, Cho/Cr, NAA/Cr, and Cho/NAA values were obtained.

Follow-up was performed after operation, and MRI scan was performed twelve months after treatment. The clinical efficacy was evaluated according to the RECIST *v* 1.0 solid tumor efficacy evaluation standard. It was considered that the complete disappearance of the lesion was a complete remission, a reduction of more than 50% of the lesion was a partial remission, a reduction of 25% to 50% of the lesion was stable, and an increase of the lesion was progress.

### 2.8. Statistical Treatments

SPSS 19.0 was used for statistical processing of experimental data. Mean ± SD was used for measurement data, and independent sample *T* test was used to compare differences between groups. The frequency (percentage) was how counting data was expressed, and chi-square test was used to compare differences between groups. *P* < 0.05 was considered statistically considerable.

## 3. Results

### 3.1. Test Results of Image Denoising Model

**Figure 3:**
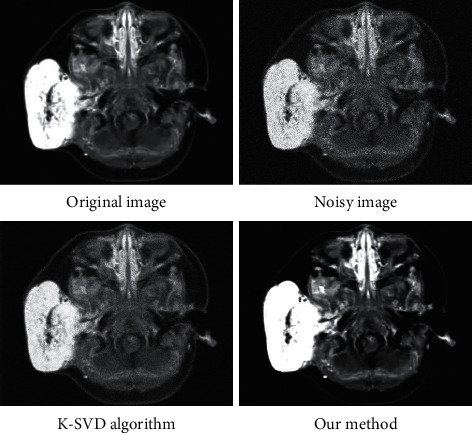
Subjective evaluation of the denoising effect of each algorithm after addition of image noise.

Noise was added to the image, and the effect of image denoising was compared with the K-singular value decomposition (K-SVD) algorithm. First, subjective evaluation of the denoising effects of different algorithms was implemented.  Figure 3 showed that the image after noise was obviously blurred. However, the image after denoising using the classic K-SVDS algorithm still had the problem of blurred details. The proposed algorithm can obviously restore the detail information in the image after image denoising, and the image definition after denoising was almost the same as the original image.

**Figure 4:**
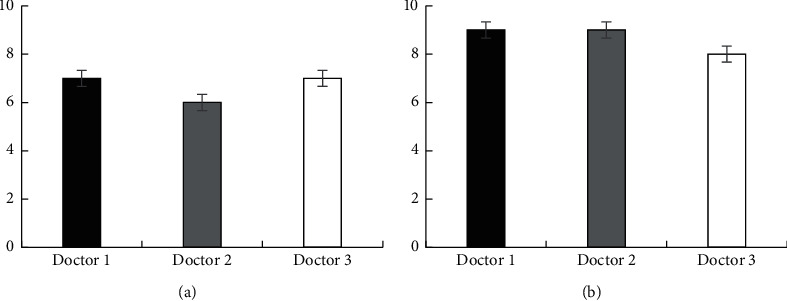
MRI image denoising effect score of the two groups of algorithms. (a) K-VSD algorithm, (b) the algorithm adopted in this study.

Doctors' evaluation of the image denoising effect obtained by the two algorithms aws shown in Figure 4. Three doctors scored 7, 6, and 7 points for the denoising effect of K-SVD algorithm, respectively, and scored 9, 9, and 8 points for the denoising effect of the algorithm, respectively. The score of the adopted algorithm was significantly higher than that of the K-SVD algorithm, and the difference was statistically significant (*P* < 0.05).

**Figure 5:**
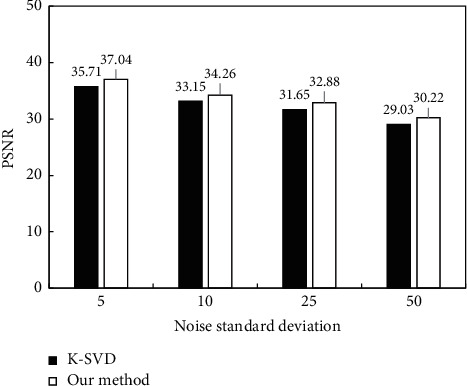
Comparison of PSNRs after algorithm denoising under different noise standard deviations.

**Figure 6:**
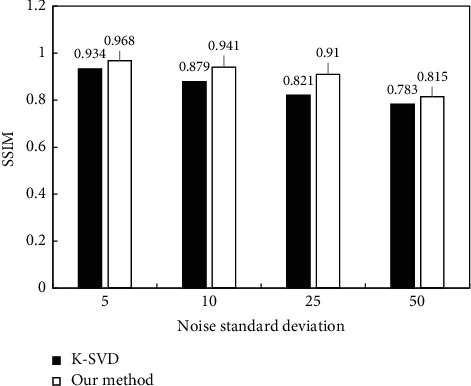
Comparison of SSIMs after algorithm denoising under different noise standard deviations.

Subsequently, PSNR and SSIM were used to quantitatively evaluate the denoising effect of each algorithm, and the results were shown in Figure 5 and 6. From Figure 5, as the standard deviation of noise in the image gradually increased, the PSNRs of K-VSD and the algorithm proposed after image denoising both showed a gradual decline. However, under different noise standard deviations, the PSNR of the image after the processing by proposed algorithm was always greater than that of the K-VSD algorithm. From Figure 6, as the noise standard deviation in the image gradually increased, the SSIM in the image after the denoising of K-VSD and the algorithm proposed also showed a gradual decline. Moreover, the SSIM of the image after the processing by the proposed algorithm denoising was always greater than that of the K-VSD algorithm.

### 3.2. Image Reconstruction Model Verification

**Figure 7:**
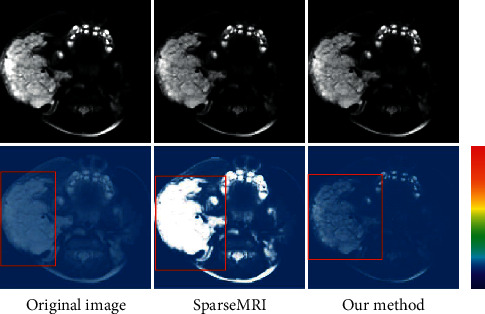
MRI image and residual image after reconstruction with different algorithms.

The common MR reconstruction algorithm Sparse MRI was set as a control, and the effect of the algorithm proposed for MRI image reconstruction was compared, to subjectively assess the difference in the effect of different algorithms for MRI image reconstruction.  Figure 7 showed that the residual image reconstructed by the proposed algorithm was obviously darker than Sparse MRI, but the detailed structure marked in the red box was remarkably better.

**Figure 8:**
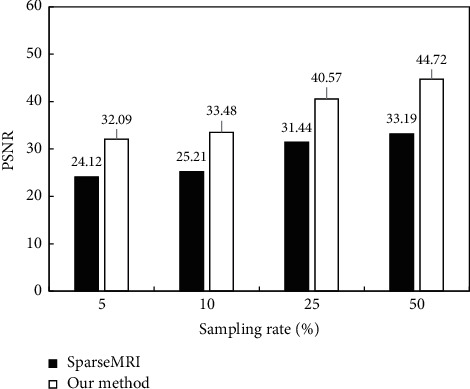
PSNR comparison after algorithm reconstruction at different sampling rates.

**Figure 9:**
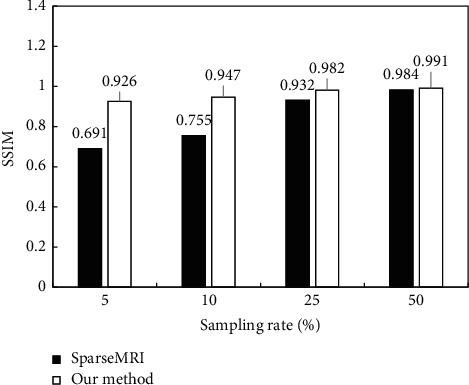
Comparison of SSIM after algorithm reconstruction at different sampling rates.

Subsequently, the difference between PSNR and SSIM in reconstructed MRI images under different random sampling rates was analyzed, and the results were shown in Figure 8 and 9. With the gradual increase of the sampling rate, the PSNR and SSIM of the Sparse MRI algorithm and the algorithm proposed for MRI image reconstruction showed a gradually increasing trend. However, under different sampling rates, the PSNR and SSIM of the proposed algorithm for MRI image reconstruction were always greater than that of the Sparse MRI algorithm.

### 3.3. Changes of MRS Metabolites in Children with SWS before and after Treatment

**Figure 10:**
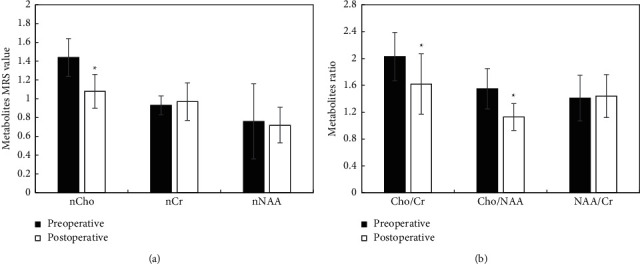
Comparison of MRS metabolite levels in the lesions of children before and after treatment. (a) the comparison of MRS metabolite level; (b) the comparison of MRS metabolite ratio.  ^*∗*^indicated that the difference between groups was considerable, (P) < 0.05.

MRS was used to detect the differences between nCho, nCr, nNAA, Cho/Cr, Cho/NAA, and NAA/Cr levels in the lesions of children with SWS before and after surgery. The results were shown in Figure 10.  Figure 10(a) showed that the level of nCho after treatment was remarkably lower than before treatment, and the difference was considerable (*P* < 0.05). There was no considerable difference between the levels of nCr and nNAA before and after treatment (*P* > 0.05).  Figure 10(b) showed that the ratios of Cho/Cr and Cho/NAA after treatment were remarkably lower than before treatment (*P* < 0.05). However, there was no considerable difference between the ratios of NAA/Cr before and after treatment (*P* > 0.05).

### 3.4. Treatment Effectiveness Evaluation

After a twelve-month follow-up, the patients suggested that there were 40 cases (88.89%) that were satisfied and improved remarkably. There were 8 cases (17.78%) of organ remodeling (displacement, difference between the thickness of the affected side and the healthy side, scar hyperplasia, and loss of detail) after the operation. There were 41 patients (91.11%) who were satisfied with the texture, color, and similarity of the transferred flaps, and there were 3 patients who wished to undergo surgery again (6.67%).

## 4. Discussion

Two algorithms were used to denoise MRI images before and after hemangiectomy for pediatric SWS, and the denoising effects of the two algorithms were analyzed. The changes of MRS metabolites and treatment effects were compared before and after the treatment. It was found that the algorithm used in this study had better denoising effect and higher PSNR and SSIM. After treatment, the levels of nCho, nNAA, Cho/Cr, and Cho/NAA of the patients were significantly reduced, and the treatment effect was obvious. Hemangioma is a relatively common benign tumor in children clinically, which seriously affects the aesthetics and related functions of children and also causes great psychological pressure on children and their parents [14]. Currently, the classification of hemangiomas is mainly based on morphological characteristics, which are mainly classified into capillary hemangioma, cavernous hemangioma, mixed hemangioma, and craniform hemangioma [15]. Clinical treatment methods for hemangioma include injection of pingyangmycin, glucocorticoid therapy, and surgical treatment [16]. Pingyangmycin has an excellent effect on hemangioma, but the treatment for superficial tumors will cause skin ulceration and pigmentation, thus affecting aesthetics [17]. Glucocorticoid therapy has poor therapeutic effect on mixed hemangioma of maxillofacial region, and skin pigmentation still exists after injection [18]. Therefore, the application of the above methods in clinical practice has certain limitations.

In this study, hemangioma resection was used for the treatment of pediatric SWS, and the appearance and function of eyes, nose, mouth, ears, and face were preserved. Moreover, MRI was used to evaluate the therapeutic effect. Studies revealed that MRI was more accurate than CT imaging in the diagnosis of hemangioma [19, 20]. However, slight movement during MR examination will cause the appearance of artifacts in MRI images [21]. MRI image processing by intelligent algorithm can improve the clinical diagnosis and treatment effect of diseases [22]. A nonlocal similar block low-rank prior algorithm was proposed to denoise and reconstruct MRI images, and PSNR and SSIM were used to evaluate the processing effect of the proposed algorithm. Currently, there are subjective evaluation methods and objective evaluation methods for the effect of algorithm processing, among which subjective evaluation mainly refers to the observation of images and evaluation of the effect of denoising, so it is prone to subjective influence and has great uncertainty [23]. PSNR and SSIM are often used in the field of image processing. When the PSNR and SSIM are larger, the processed image is more similar to the original image, and the structural information is better preserved [24, 25]. The nonlocal similar block low-rank prior algorithm was used for MRI image denoising and reconstruction, which was compared with the classical denoising algorithm K-SVD [26] and reconstruction algorithm Sparse MRI [27]. It was found that the PSNR and SSIM of the image processed by the proposed algorithm were large, indicating that the MR image effect processed by the nonlocal similar block low-rank prior algorithm was ideal, which was beneficial for doctors to extract information from MRI images.

Subsequently, MRS was used to evaluate the tissue biochemistry and functional metabolism of the lesion before and after hemangioma resection treatment. The main metabolites detected by MRS included Cho, NAA, and Cr. Cho is one of the main complexes constituting the cell membrane, which participates in the synthesis and catabolism of the cell membrane [28]. NAA mainly exists in the cytoplasm of neurons. It is a marker of neuronal activity and density. When neuronal function is lost or dies, NAA levels decrease [29]. Cr is closely related to energy metabolism [30]. In this study, the levels of Cho, NAA, and Cr, the ratio of Cho/NAA, Cho/Cr, and NAA/Cr in the lesions of the children before and after treatment were detected. The results showed that the standardized Cho level after treatment, as well as the Cho/Cr and Cho/NAA ratios, was remarkably reduced. Some studies suggested that Cho can be used to assess tumor proliferation after certain treatment, and the ratio of Cho/Cr and Cho/NAA can increase with the increase of tumor malignancy [31]. In this study, MRI images were used to evaluate the treatment efficiency, and the results showed that the improvement rate of hemangioma resection treatment was as high as 88.89%. It was proved that after hemangioma resection to treat pediatric SWS, the tumor tissue and its residue can be accurately removed. The limitation of this study is that MRI images are only used to evaluate the therapeutic effect of vasectomy, and the evaluation method is too simple. More evaluation indicators should be explored later in order to comprehensively and accurately evaluate the therapeutic effect.

## 5. Conclusion

The MRI image denoising effect of the algorithm adopted in this study was relatively better, the doctor score was higher, and the PSNR and SSIM were higher relative to those traditional methods. After treatment, the patient's treatment effect was ideal. The proposed algorithm is worth promoting and applying actively in clinic.

## Data Availability

The data used to support the findings of this study are available from the corresponding author upon request.
